# CXCL12 Chemokine Expression Suppresses Human Pancreatic Cancer Growth and Metastasis

**DOI:** 10.1371/journal.pone.0090400

**Published:** 2014-03-04

**Authors:** Ishan Roy, Noah P. Zimmerman, A. Craig Mackinnon, Susan Tsai, Douglas B. Evans, Michael B. Dwinell

**Affiliations:** 1 Department of Microbiology and Molecular Genetics, Medical College of Wisconsin, Milwaukee, Wisconsin, United States of America; 2 Department of Pathology, Medical College of Wisconsin, Milwaukee, Wisconsin, United States of America; 3 Department of Surgery, Medical College of Wisconsin, Milwaukee, Wisconsin, United States of America; University of Florida, United States of America

## Abstract

Pancreatic ductal adenocarcinoma is an unsolved health problem with nearly 75% of patients diagnosed with advanced disease and an overall 5-year survival rate near 5%. Despite the strong link between mortality and malignancy, the mechanisms behind pancreatic cancer dissemination and metastasis are poorly understood. Correlative pathological and cell culture analyses suggest the chemokine receptor CXCR4 plays a biological role in pancreatic cancer progression. *In vivo* roles for the CXCR4 ligand CXCL12 in pancreatic cancer malignancy were investigated. CXCR4 and CXCR7 were consistently expressed in normal and cancerous pancreatic ductal epithelium, established cell lines, and patient-derived primary cancer cells. Relative to healthy exocrine ducts, CXCL12 expression was pathologically repressed in pancreatic cancer tissue specimens and patient-derived cell lines. To test the functional consequences of CXCL12 silencing, pancreatic cancer cell lines stably expressingthe chemokine were engineered. Consistent with a role for CXCL12 as a tumor suppressor, cells producing the chemokine wereincreasingly adherent and migration deficient *in vitro* and poorly metastatic *in vivo*, compared to control cells. Further, CXCL12 reintroduction significantly reduced tumor growth *in vitro,* with significantly smaller tumors *in vivo*, leading to a pronounced survival advantage in a preclinical model. Together, these data demonstrate a functional tumor suppressive role for the normal expression of CXCL12 in pancreatic ducts, regulating both tumor growth andcellulardissemination to metastatic sites.

## Introduction

Pancreatic ductal adenocarcinoma (PDAC) is anunrelenting form of human cancer, with no effective technique for early diagnosis or treatment, and an incidence nearly equal to mortality [1], [2]. Most patients, at the time of diagnosis, already have locally advanced or metastatic disease, making surgical resection of the primary tumor of limited therapeutic value [3].Current pharmaceutical therapies for PDAC are inadequate as the vast majority of pancreatic cancer patients die of micro- or macro-metastatic disease [4]. An improved understanding of the biologic mechanisms underlying the aggressiveness of PDAC is needed. Although recent studies have determined the molecular factors involved in the progression of PDAC [5]–[7], little is known about the biologic mechanisms regulating cell motility and, in turn,metastasis. Chemotactic cytokines, or chemokines, play keyroles in cellular migration, and are capable of coordinating multiple aspects of the cell migration machinery. Through activation of their receptors, chemokinesregulate several facets of normal physiology, including leukocyte trafficking, epithelial cell migration necessary for wound closure, tissue vascularization, and organ development during embryogenesis [8]–[10]. Previously, our lab revealedthe importance of the dual expression of the chemokine CXCL12 and its cognate receptor CXCR4 in enterocyte migration, wound healing, and angiogenesis [11]–[16].

Several studies have implicated a role for chemokine receptors, in particular CXCR4 and CCR7, in cancer progression and metastasis [9], [17]–[22]. CXCR4 expression has been linked with the malignancy of over 20different cancers [17], [23]. We have previously demonstrated that CXCL12 is expressed in normal epithelial cells in intestine and mammary glands, but is epigenetically silenced in human breast and colorectal cancer [24], [25].This pattern of CXCL12 gene repressionresults in tumor cells whose chemokine – receptor profilesmirror that of highly mobile leukocytes, which express the receptors CXCR4 and CXCR7 but not the ligand. We showed that re-expression of CXCL12 decreased the ability of breast and colorectal cancer cells to metastasize [24]–[26].Several studies haveextended those seminal findings to show beneficial effects of autocrine CXCL12 in breast, lung, gastric, and head and neck cancers [27]–[30]. Loss of CXCL12expression in osteosarcoma and breast cancer patient tumor specimenswas correlated with poorer prognosis [31]–[34]. In pancreatic cancer, conflicting and incomplete reports suggest either that CXCL12 expression is elevated or that CXCL12 is not expressed in the vast majority of patients [35], [36]. Thus while a growing body of evidence suggests a tumor-suppressive role for CXCL12 in human cancer malignancy, its mechanistic roles in PDAC remain poorly understood.

The prevailing model of cancer metastasis is that malignant tumors spread to distant tissues through the over-expression of CXCR4 [22], [37], [38], thereby sensitizing malignant cells to spread in response to distant gradients of CXCL12 produced by metastatic destination organs. This model has also been used to explain PDAC metastasis, as several reports document the expression of CXCR4 by pancreatic carcinoma cell lines and human tissue [39]–[42]. However none of these studies have rigorously examined the parallel expression of CXCL12 or CXCR7 in the context of CXCR4 or defined expression of any of the three components of this chemokine axis in normal pancreatic epithelium.The objectives of our study were to determine the expression profile and functional role of the CXCL12-CXCR4-CXCR7 axis in both healthy normal and malignant pancreas. Herein, our data demonstrate that while receptor expression is increased,CXCL12 expression is significantly diminished in resected PDAC tissue and a battery ofpancreatic cancer cell lines compared to normal pancreata. CXCL12 expression was transiently restored using inhibitors of epigenetic repression.Stable reintroduction of CXCL12 in PDAC cells prevented directed cell migration and hepatic metastasis and slowed tumor growth*in vitro* and*in vivo*, resulting in increased survival in preclinical models.

## Materials and Methods

### Ethics statement

Exempt, de-identified normal pancreata, PDAC tissue specimens, and unique patient-derived PDAC cells were obtained from the Surgical Oncology Biorepository using a Medical College of Wisconsin (MCW) InstitutionalReview Board #4 approved protocol. Tissues and cells within the biobank were obtained from patients following signed written informed consent and are coded and de-identified,with personal identifying information not shared with research investigators. Preclinical mouse xenograft studies were completed using protocols and procedures documented in an established Animal Use Application(AUA076) approved by the MCW Institutional Animal Care and Use Committee and in compliance with guidelines established by Office of Laboratory Animal Welfare and in accordance with Guide for the Care and Use of LaboratoryAnimals. All mice were housed under pathogen-free conditions, received food and water ad libitum, and maintained in a 10hr:14-hr light/dark cycle in a temperature-controlled room (25±2°C).Animals were euthanized at the completion of the study or following signs of distress or poor body condition as assessed by trained laboratory personnel and confirmed by MCW Biomedical Resource Center staff veterinarians to ameliorate pain and distress associated with tumor formation.

### Human pancreatic cancer cell lines

Panc1(CRL-1469), MiaPaCa2(CRL-1420), Capan2 (HTB-80), and HPAFII (CRL-1997) cell lines were purchased from the ATCC. Panc1 and MiaPaCa2 cell lines were maintained in DMEM supplemented with 10% (v/v) fetal bovine serum (FBS). The Capan2and HPAFII cell lines were maintained in 10% (v/v) FBS supplemented McCoy's 5Aor MEM medium, respectively. In some experiments, cells grown in normal medium were treated daily with 5-aza-2′-deoxycytidine (5-aza) or Trichostatin-A (EMD Biosciences). MiaPaCa2 cell lines were transfected with Firefly-luciferase under zeocin selection. Resultant luciferase-expressing MiaPaCa2 cells were subsequently transfected with plasmids encoding either eGFP or human CXCL12, and clones grown under neomycin selection conditions [26]. Cell lines were de-identified of all identification parameters from individual consenting patients with pancreatic cancer and were confirmed to be of PDAC origin following DNA karyotype and protein expression analysis.

### Human pancreatic tissuespecimens

Normal ducts and PanIN lesions were isolated exclusively from adjacent normal tissue discards of organ transplantation or pancreatic operations not involving PDAC or other exocrine malignancies. Disease status of those normal and tumor tissueswas confirmed by a board-certified pathologist.A total of 25 tissues from 21 different patients were used for normal analyses. A total of 82 tissues from 29 different patients were used for tumor analyses. Of the 29 patients providing PDAC tissue, 11 patients had received neo-adjuvant therapy.

### RT-PCR

RNA was isolated from cells and tissue specimens using the RNAeasy kit (Qiagen) and treated with DNase (Ambion), converted to cDNA, and CXCL12, CXCR4, CXCR7, and GAPDH transcript expression analyzed as defined and previously optimized for efficiency in a linear range [24], [43].A region of the 5′-untranslated region of the mannose binding lectin gene was amplified to detect genomic DNA contaminants [12].

### Immunoanalyses

Unstained slides were generated from pancreatic tumor specimens and CXCL12 immunostained using an antibody from R&D Systems (mab350). Cytokeratin-19 (CK19) (ab7754), CXCR4 (ab2074),CXCR7 (ab38089 & ab72100), and Ki-67 (ab15580) were detected using antibodies from Abcam.Visualization was done using horseradish-peroxidase-conjugated secondary antibodies and the 3,3′-diaminobenzidinePeroxidase Substrate Kit(Vector Labs). To avoid background interference slides were not counterstained. Protein expression levels were scored using a standard 4-point scale [0 = absent, 1 = weak, 2 = mixed, and 3 = strong staining intensity] [44]. Analyses were independently completed by an investigator blinded to the disease status and immunostaining protocol. Secreted CXCL12 protein from supernatant of pancreatic cancer cells cultured in serum-free media was detected by ourpreviously established sandwich ELISA method using antibodies from R&D Systems (monoclonal mouse and human CXCL12 (MAB350) and goat anti-human CXCL12 (BAF310) [24].Cell surface CXCR4 or CXCR7was detected using the aforementioned antibodies (Abcam) along withFITC-conjugated secondary antibodies using our previously established method [43]. Briefly, cells were cultured in normal growth medium, lifted using enzyme-free dissociation buffer, washed using ice-cold sterile PBS, and then incubated in a blocking solution containing BSA and secondary antibody serum. After blocking, cells were first incubated with primary antibody and then with FITC-conjugated secondary antibody. Finally, cells were fixed and analyzed by flow cytometry (LSR II, BD Biosciences).

### *In vitro* tumorigenesis assays

Cells were plated to the upper well with chemo-attractants added to the bottom well and transwell migration or chemoinvasion enumerated in a representative set of images taken after fluorescent staining as described previously [43], [45]. The upper well membrane was coated with collagen in migration assays and with Matrigel (BD Biosciences) in invasion assays.Panc1 and HPAFII cell migration was measured after 24 hour stimulation in transwells while MiaPaCa2 cell migration was measured after 6 hours.

Initial proliferation and apoptosis of PDAC cell lines was defined using the Viacount flow cytometric assay (Millipore),or the caspase-3/7 glo assay (Promega).Briefly, cells were plated in 10% (v/v) serum-containing medium, and once adherent (overnight) were switched to serum-free medium. Cell cycle analysis was done using propidium iodide staining and flow cytometric analysis, as done previously [43]. In some experiments cells were grown in 1% (v/v) serum-containing media after 24 hours of serum starvation.Gemcitabine (GEM), a well-established chemotherapeutic drug in pancreas cancer patients,was used as a positive control for decreased growth and increased apoptosis.

### *In vivo* studies and bioluminescence imaging

An established heterotopic intrasplenic injection model [46]was usedto assess metastatic homing and extravasation in the liver. Six-week-old immunocompromised SCID mice were anesthetized and 1×10^6^MiaPaCa2luciferasecells were injected into the spleenthrough a lateral wall excision and tumor growth and metastasis monitored using bioluminescence imaging every 7 days using a previously described approach [26]. After 28 days, mice were sacrificed and tumor formation in the spleen and liver measured *ex vivo* using bioluminescence imaging. An orthotopic model was employed as previously established [47], with 1×10^6^ cells injected into the pancreata of SCID mice and disease progression monitored. Mice were removed from the study when thetumor size reached 1000 mm^3^ volume and 1×10^9^p/sec/cm^2^/steradianradiance.Three mice engrafted with CXCL12-expressing cells were removed for veterinary non-study reasons due to cage-infighting.

### Statistical analyses

Multiple comparisons between groups were analyzed using a one-way ANOVA and a Dunnett *post-hoc* analysis used to identify pair-wise differences (GraphPad Prism 4). Paired analyses were calculated using either a Mann-Whitney or log-rank test where appropriate. Statistical significance was defined as *P*≤0.05.

## Results

### Reciprocal CXCL12, CXCR4, and CXCR7 expression in pancreatic ductal adenocarcinoma

**Figure 1:**
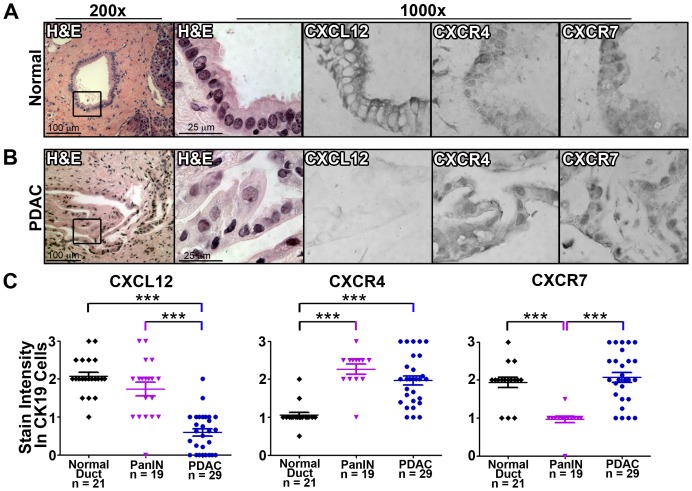
Aberrant chemokine ligand and receptor expression in human pancreatic ductal adenocarcinoma. Serial sections of normal and diseased pancreatic tissue were stained for CXCL12, CXCR4, CXCR7, and CK19. CXCL12 staining apparent in normal exocrine ducts was diminished in PDAC tissue. CXCR4 staining increased in PanIN and PDAC relative to normal ductal epithelium. CXCR7 expression was variable in normal epithelium, PanIN lesions, and PDAC. (A)Normal tissue from a single patient with healthy pancreas represents observations from 25 different normal tissues from 21 individual patients. (B)PDAC tissue from one patient represents observations from 82 different tissues from 29 different patients. 1000× magnification represents inset box at 200×. (C) Staining was quantified by blinded scoring of serial sections in relation to CK19 staining. (***) denotes *P*≤0.001.

**Figure 2:**
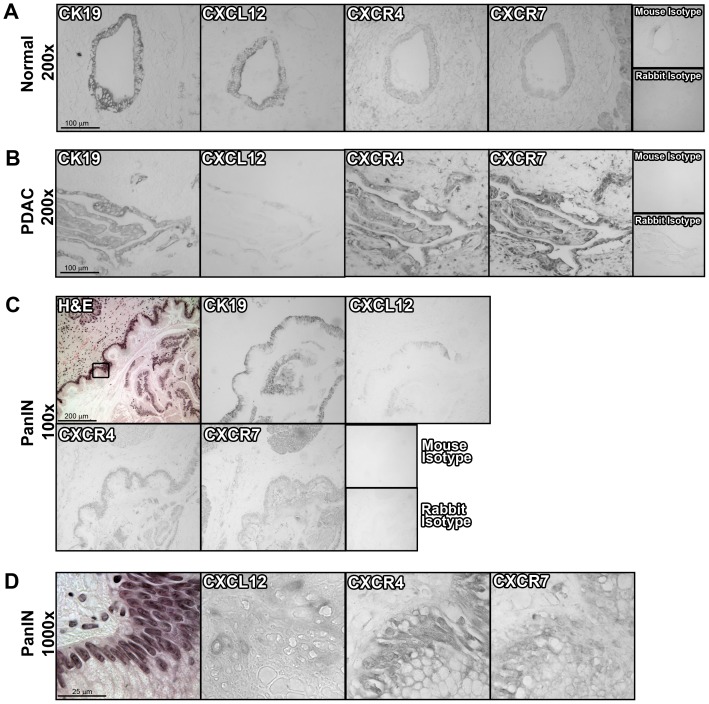
CXCL12 expression in normal pancreas is decreased in dysplastic epithelium. Representative 200× images of the same (A) normal or (B) pancreatic ductal adenocarcinoma (PDAC) tissues shown at 1000× magnification in Figure 1. Representative serial section images of a pancreatic intestinal neoplasm (PanIN) lesion at (C) 200× or (D) 1000× magnification. Serial tissue sections were immunostained with antibodies to CK19, CXCL12, CXCR4, and CXCR7, or the isotype controls. n  =  25 different normal tissues from 21 individual patients, 82 different tissues from 29 different PDAC patients, or 19 pathologically confirmed PanIN lesions.

The functions of CXCR4, CXCR7 or CXCL12 in pancreatic cancer progression remain based on limited analyses without simultaneous analysis of all three chemokine signaling components in both normal and diseased tissue.Immunostaining of serial tissue sectionswith specific antibodies,revealedrobust CXCL12 staining on normal ductal epithelial cells, with those same epitheliaalso staining positive for CXCR4 and CXCR7(Fig.1A &Fig. 2A). CXCL12 chemokine expression was specifically restricted to the ductal compartment and absent from acinar and endocrine cells, both of which stained for the chemokine receptors CXCR4 and CXCR7 (data not shown). Incubation of parallel sections with isotype control antibodies confirmedspecificity (Fig. 2A). We next examined expression in pre-malignant pancreatic lesions, known as Pancreatic Intra-epithelial Neoplasms (PanINs) [48]–[51]. As shown in Figure 2C-D, CK19+ PanINsexpressed both CXCR4 and CXCR7. By comparison, CXCL12 staining wasmore variable, with mixed tomarkedly decreased expression in PanINs compared to normal pancreatic ducts (Fig.2C–D). In a limited analysis, no significant differences in CXCL12 expression could be detected between PanIN1, PanIN2, and PanIN3 lesions.

Analysis of PDAC tumor tissue revealeda further, and more pronounced,extinction of CXCL12 in CK-19+tumor cells (Fig.1B, Fig.2B). In heterogeneous tumor tissue containing both malignant and normal glands, CXCL12 staining was diminished in malignant cells,butpreserved in adjacent normal ductal cells (data not shown). Analysis of serial tissue sectionsrevealed that malignant tissue with low levels of CXCL12had a reciprocal increase in CXCR4, as well as CXCR7, staining (Fig. 1B, Fig. 2B). Quantification of the CXCL12, CXCR4, and CXCR7 immunostaining intensity confirmed the significant decrease in CXCL12 expression during progressionfrom normal to precursor PanIN to malignant pancreatic tissue (Fig. 1C). In contrast, CXCR4 expression was elevated in PanINsand PDAC compared to normal ducts (Fig.1C). Scoring of CXCR7 expression revealed a biphasic pattern with lower expression occurring at the PanIN stage and higher expression recurring in PDAC (Fig.1C).When the patients were subdivided into groups dependent on whether or not they had received neo-adjuvant therapy, patients receiving standard-of-care regimens of gemcitabine or FOLFIRINOX therapy[52]–[54] had significantly (*P*≤0.05) higher scores for CXCL12 expression (0.82±0.18) compared to patients not receiving neoadjuvant therapy (0.43±0.09).

### CXCL12 expression and epigenetic regulation in pancreatic cancer cell lines

**Figure 3:**
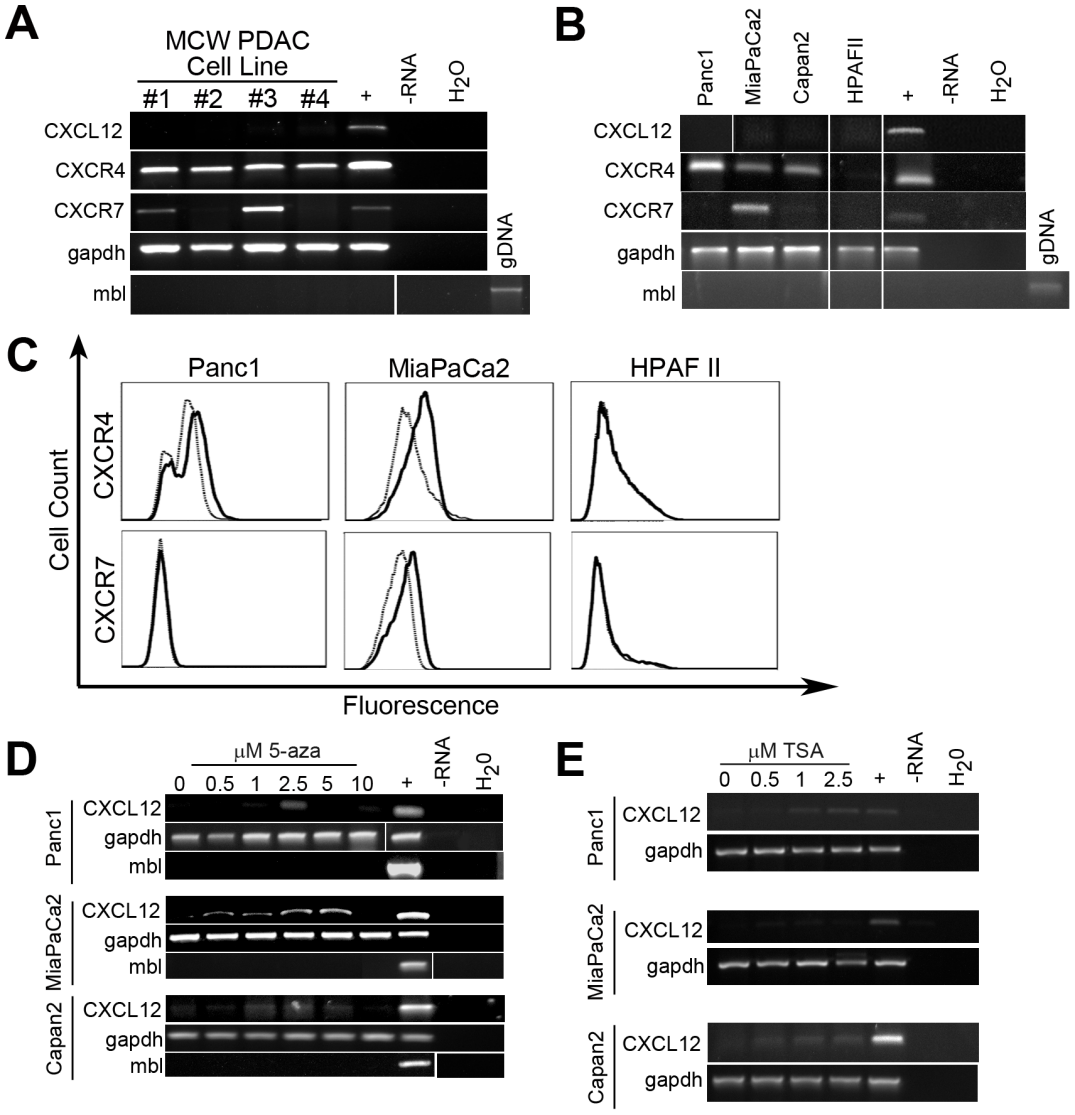
CXCL12 expression in human pancreatic ductal adenocarcinoma cell lines. RT-PCR analysis revealed that (A) patient-derived pancreatic cancer cell lines (#1, #2, #3, #4) or (B) established cell lines lacked expression of CXCL12 and maintained expression of CXCR4. CXCR7 mRNA was present in 3 of 8 PDAC lines.Flow cytometric detection(C)of surface CXCR4 or CXCR7 protein expression. Cell lines treatedseven days with a concentration curve of (D) 5-aza-2-deoxycytidine (5-aza) restored expression of CXCL12.Linestreated 4 days with a concentration curve of (E) Trichostatin-A (TSA)restored CXCL12 mRNA expression in Capan2 cells.

To further characterize chemokine – receptor expression and identify an appropriate model for studying the function of CXCL12 in PDAC,primary patient-derived and established pancreatic cancer cell lines were analyzed through RT-PCR using our previously optimized primer sets for CXCL12, CXCR4, and CXCR7 [24], [43]. RT-PCR indicated consistent lack of CXCL12 transcript in patient-derived primary as well as Panc1, MiaPaCa2, Capan2, and HPAFII cell lines (Fig.3A–B). In agreement with the immunohistochemical analyses, CXCR4 mRNA was maintained in 7of the 8 PDAC cell lines, while CXCR7 expression was more variable (Fig.3A–B). Flow cytometry confirmed the cell-surface localization of CXCR4 and CXCR7 on representative cell lines (Fig.3C). To ascertain if the lack of CXCL12 expression reflected epigenetic silencing, cells were treated with titrated doses of the DNA methyltransferase inhibitor 5-aza. The inhibitor rescued CXCL12mRNA expression in representative cell lines (Fig.3D). Treatment with the histone deacetylase inhibitor Trichostatin-Aalso restored expression of CXCL12 in Capan2 cells(Fig. 3E). Based upon our prior investigations examining the specific mechanisms of CXCL12 promoter hypermethylation in colorectal and breast cancers [24], [25], these data suggest that CXCL12 expression is regulated through epigenetic mechanisms in pancreatic cancer as well.Cumulatively, thesedata indicate CXCL12 gene repression in human tissueand a battery of new and established PDAC cell lines.The MiaPaCa2 cell line was identified as an ideal model, as its CXCL12-CXCR4-CXCR7 expression pattern mimics that observed in malignant human PDAC tissue.

### CXCL12 re-expression in human PDAC cells disruptedchemotaxis, increased adhesive potential, and decreased hepatic metastasis

**Figure 4:**
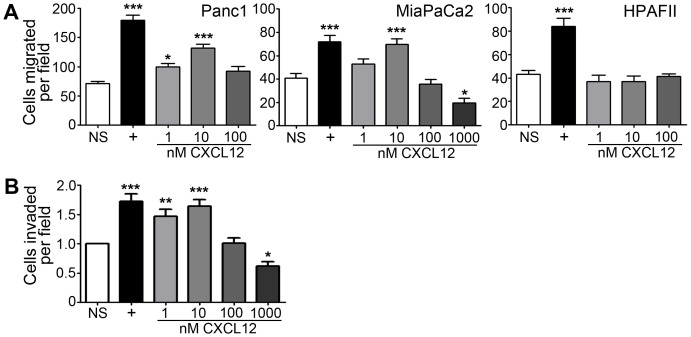
PDAC cells migrate and invade following acute exogenous CXCL12 stimulation. (A) Panc1 and MiaPaCa2 cells migrated towards exogenous gradients of CXCL12 in a range from 1 nM to 1000 nM under serum-free conditions(*), (**), and (***) denote *P*≤0.05, *P*≤0.01, and *P*≤0.001, respectively, in comparison to unstimulated cells (NS). Receptor-null HPAFII cells did not migrate in response to CXCL12. (B) CXCL12 directs Panc1 cell chemoinvasion into a three-dimensional Matrigel plug. The positive control (+) was 10% serum-containing medium. (*), (**), and (***) denote *P*≤0.05, *P*≤0.01, and *P*≤0.001, respectively, in comparison to unstimulated cells (NS).

The conventional paradigm for chemokine mediated metastasis of cancer cells is that expression of CXCR4 is pro-metastatic. This model stems from the ability of exogenous CXCL12 to stimulate migration of cancer cells *in* vitro [13], [45], [55]. As expected, measurement of the native migration potential of several CXCL12-deficient pancreatic cell lines revealed that CXCR4-expressing PDAC cells migrate towards acute CXCL12 stimulation (Fig. 4A). Importantly, Panc1 and MiaPaCa2 cells migrated towards CXCL12 in biphasic-concentration dependent manner consistent with current understanding of chemotactic migration [55], [56]. The HPAFII cell line which lacked surface expression of either CXCR4 or CXCR7 was unable to migrate in response to CXCL12 treatment (Fig. 4A). Panc1 cellsalso invaded an extracellular matrix in response to acute exogenous CXCL12 stimulation (Fig. 4B).

**Figure 5:**
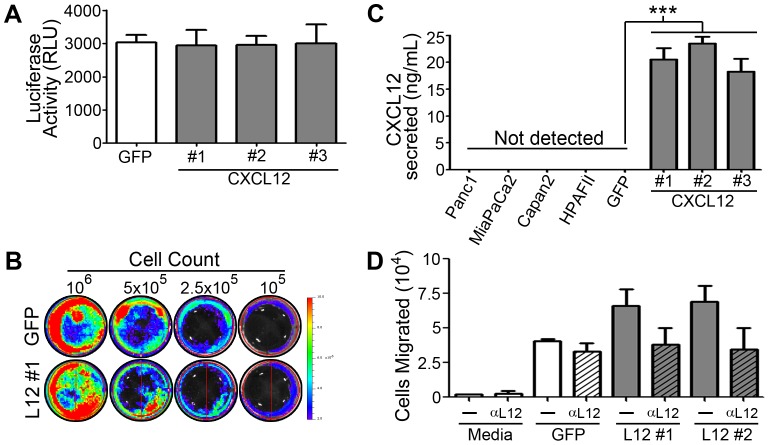
Double transfectant Firefly luciferase and GFP- or CXCL12-expressing MiaPaCa2 cells. Luciferase levels in control GFP or three different (31, #2, #3) CXCL12-expressing MiaPaCa2 clones as measured by spectrophotometer (A) or IVIS-100 biophotonic imager (B). Levels of CXCL12 were measured by ELISA (C) in established PDAC cell lines as well as GFP- and CXCL12-expressing MiaPaCa2-luciferase clones. (D) CXCL12-secreted by transfected MiaPaCa2-luciferase clones #1 and #2 stimulated U937 chemotaxis. Cells treated with neutralizing antibody to CXCL12 (αL12) confirmed the specificity of U937 chemotaxis. Values in A, C, and D are mean±SEM, n = 2–3.

**Figure 6:**
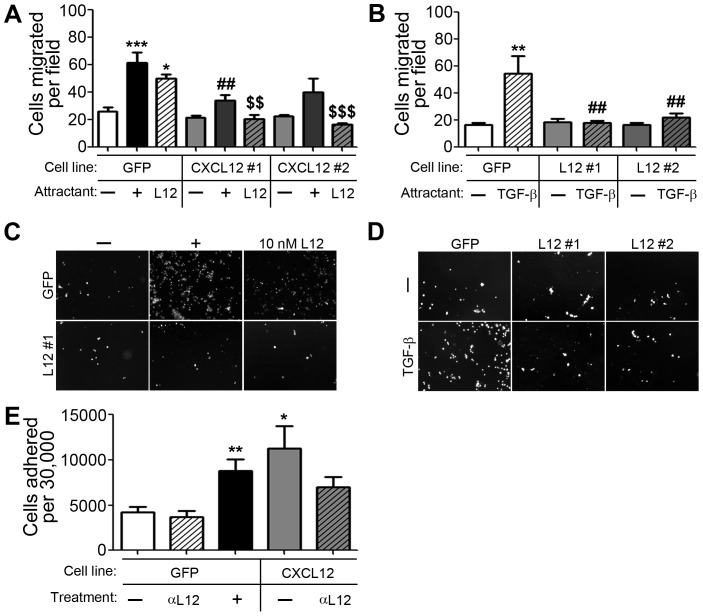
Chronic CXCL12 production decreased migrationpotential and increased cell:substrate adhesion. (A) Transwell migration assays revealed significantly reduced chemotaxis of CXCL12-expressing clones (#1 and #2) compared to ligand null (GFP) cells. Attractants were serum-free media (--), 10% serum (+), or 10 nM CXCL12 (L12) in serum-free media. 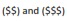 denote *P*≤0.01 and *P*≤0.001, respectively, compared to 10 nM CXCL12-stimulated GFP cells, n = 5. (B) CXCL12 re-expression diminished TGF-β (5 ng/mL)-induced chemotaxis relative to the CXCL12-null cells. (##) denotes *P*≤0.01 compared to TGF-β-stimulated GFP cells, n = 4. (C) & (D) Representative images of experiments in (A) and (B) respectively. (E) CXCL12-expressing cells were significantly more adherent to tissue culture plastic compared to CXCL12-null cells. Untreated cells  =  (--), neutralizing antibody for CXCL12 activity  =  (αL12), and a positive control  =  1 ng/mL of EGF (+). (##) denotes *P*≤0.01 compared to 10% serum-stimulated control cells. (*), (**), and (***) denote *P*≤0.05, *P*≤0.01 and *P*≤0.001, respectively, compared to unstimulated GFP cells, n = 7.

We believe that the pro-metastatic response of PDAC cells is due to the silencing of expression of CXCL12. To test this were-introduced expression of the chemokine, using doublestable plasmid integration, into MiaPaCa2 cells. Cells were first stably transfected with firefly-luciferase and then transfected with additional genes using a second selection reagent.Several CXCL12-expressing clones were generated along with a control clone transfected with eGFP, each exhibiting equivalents levels of luciferase activity (Fig.5A–B). Importantly,lack of chemokine production was confirmedby ELISA in Panc1, MiaPaCa2, Capan2, and HPAFII cell lines (Fig. 5C). Functional chemokine production in CXCL12-expressing clones compared to GFP-expressing clones to of MiaPaCa2 cells was validated by ELISA andtranswell migration of U937 cells (Fig.5C–D).MiaPaCa2 cells secreting CXCL12demonstrated a significant reduction in migratory response to TGF-βor CXCL12, both known drivers of *in vitro* PDAC cell migration (Fig.6A–D). Given the importance of adhesive potential in the metastatic abilities of cancer cells, cell adhesion was next measured. CXCL12 positive cells were significantly more adherent to tissue culture plasticcompared to chemokine null cells (Fig. 6E). These *in vitro* data indicate that the overall malignant potential of pancreatic cancer cells,both in the ability of these cells to migrate and evade substrate adhesion, is decreased following reintroduction of the CXCL12 transcript into PDAC cells.

**Figure 7:**
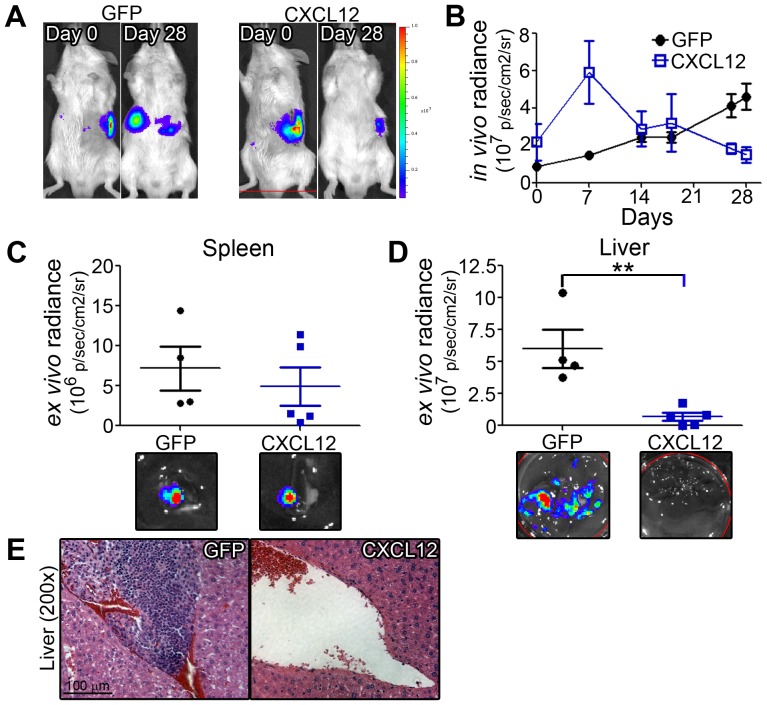
Decreased liver metastasis of circulating CXCL12-expressing PDAC cells. (A) Representative bioluminescence images of mice xenografted with GFP- or CXCL12-expressing cells at implantation (Day 0) or study endpoint (Day 28). (B) Whole-body *in vivo* radiance over time of GFP- and CXCL12-mice. (C) *Ex vivo* radiance of excised spleen (C), reflecting tumor cells at the site of injection, or the metastatic destination (D), reflecting decreased hepatic metastasis of CXCL12-expressing cells relative to GFP-cells. (**) denotes *P*≤0.01, n = 4–5. (E) Representative H&E images showing pronounced tumor mass in the liver of control (GFP), relative to experimental (CXCL12) xenografted mice.

We next sought to determine whether the simultaneous increased adhesive and decreased migratoryphenotypes in PDAC cells would suppressmetastatic spread*in vivo* usinga heterotopic xenograft mouse model.CXCL12-expressing and CXCL12-null cells were injected into the spleen of SCID mice. Over 28 days,*in vivo* tracking of cells indicated that, compared with control cells, there was a significant alteration in the ability of CXCL12-expressing MiaPaCa2 cells to disseminate away from the initial site of inoculation (Fig.7A–B). *Ex vivo* analysis indicated that luminescence at the site of splenic inoculation was unchanged, while mice injected with PDAC that produce CXCL12had significantly lower hepatic tumor burden than mice injected with GFP-control cells (Fig.7C–E). These data suggest that CXCL12 specifically alters the ability of pancreatic cancer cells to home to the liver, an organ with high expression of CXCL12 and a common metastatic destination of PDAC patients.

### Concomitant CXCL12, CXCR4 and CXCR7 expression decreased tumor cell proliferation and metastasis and prolonged survival in a preclinical PDAC model

**Figure 8:**
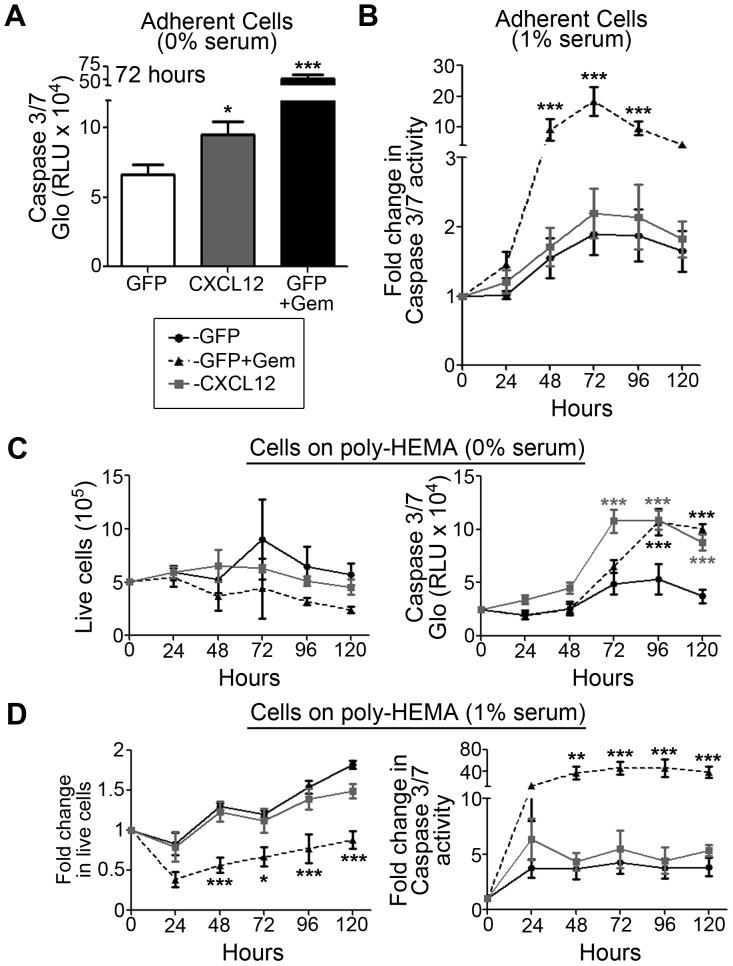
CXCL12-positive pancreatic cancer cells have minimal change in adherent apoptosis or anoikis. Apoptosis of GFP and CXCL12-expressing MiaPaCa2 cells were assessed using the caspase 3/7 glo assay.Cells were starved for 24 hours and cultured in 0% serum (A, C) or 1% serum (B, D) containing medium. (A–B) Apoptosis in adherent CXCL12-expressing cells was elevated compared to either GFP-expressing clones in serum-free conditions with no change seen in 1% serum. GFP-cells werestimulated with 100 µM gemcitabine as a control. (C–D) To measure cell number and detachment based apoptosis of cells in suspension, MiaPaCa2-luciferase cells were cultured on poly-HEMA. Using the Viacount reagent and flow cytometric cell counting, there was no difference in live cell number observed in non-adherent CXCL12-null (GFP) or expressing cells when cultured either in 0% (C) or 1% serum (D). Apoptosis of poly-HEMA cultured cells revealed an in increase in active caspase-3/7 restricted to cells cultured in serum-free conditions only (C) with no change observed in 1% serum (D). Gemcitabine (GEM) was used as a control for decreased cell count and increased apoptosis.(*), (**), and (***) denote *P*≤0.05, *P*≤0.01, and *P*≤0.001 respectively in comparison to control cells (GFP). Values are mean±SEM, n = 4–5.

Previously, we had observed that re-expression of CXCL12 in colorectal cancer cells lead to increased anoikis, detachment based apoptosis [24], [43]. We tested whether re-introduction of CXCL12 into PDAC cells would change their apoptotic or proliferative potentials. In serum-free conditions,an increase in apoptosis was detected in adherent CXCL12-expressing pancreatic cancer cells when compared with GFP-expressing controls, though this response was attenuated with the addition of serum (Fig. 8A–B). Using an established model for studying detachment-induced apoptosis, cells were cultured on Poly-HEMA [43]. As with adherent cells, apoptosis was increased in non-adherent CXCL12-positive PDAC cells compared to controls, but again the addition of serum resulted in no change in apoptosis(Fig.8C–D). Cell number remained unchanged between non-adherent CXCL12-positive and negative cell populations (Fig. 8C–D).

**Figure 9:**
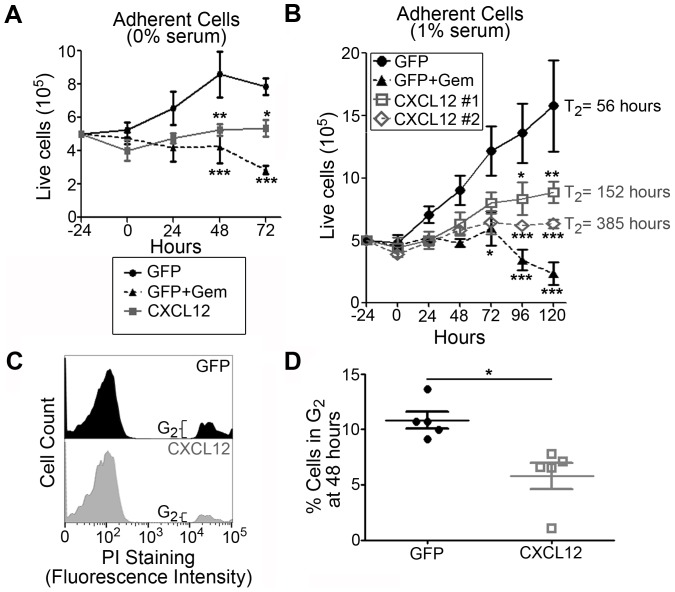
CXCL12 expression results in decreased proliferation of PDAC cells. Population growth of adherent GFP and CXCL12-expressing MiaPaCa2 cells was assessed using the Viacount reagent and flow cytometric cell counting (A–B). CXCL12-expressing cells starved for 24 hours and cultured in both 0% serum (A) or 1% serum (B) containing medium were found to have decreased population growth.(B) Two CXCL12-expressing clones (#1, #2) were compared to GFP alone or GFP + gemcitabine (GEM) controls in 1% serum containing medium. Doubling time of adherent clones (T_2_) was calculated using a linear regression of the data to determine slope and the intercept at y = 10^6^, with increased T_2_ observed in both CXCL12-expressing clones. (C–D) Propidium Iodide cell cycle analysis revealed a decrease in percentage of cells in the G_2_ phase in CXCL12-expressing cells compared to GFP controls. (*), (**), and (***) denote *P*≤0.05, *P*≤0.01, and *P*≤0.001 respectively in comparison to control cells (GFP). Values are mean±SEM, n = 4–5.

Given the minimal change in anoikis-sensitivity in CXCL12-expressing PDAC cells in serum containing conditions, we next tested their growth potential. In marked contrast to our data in human colorectal or breast cancers [24]–[26], [43], population growth of CXCL12 expressing PDAC was significantly decreased compared to chemokine deficient cells (Fig.9). Slower growth of CXCL12-expressing cells was observed in both serum-free and serum-containing conditions (Fig.9A–B). Decreased population growth was restricted to adherent cells as there was no measurable change in the number of cells detected in the supernatant of either population (data not shown).Two separate CXCL12-expressing clones demonstrated doubling times significantly greater than the doubling time observed in GFP-expressing cells (Fig. 9B).

We hypothesized that the decrease in growth potential from forced CXCL12 expression was due to cell cycle arrest. Using propidium iodide staining, we measured a ∼50% decrease in the percent of CXCL12-expressing pancreatic cancer cells in the G2 phase of cell cycle, compared to GFP-expressing controls (Fig. 9C–D). These data, along with the cell counting study, suggest that CXCL12-expressing cells have decreased proliferation related to cell cycle arrest prior to entering the G2phase.

**Figure 10:**
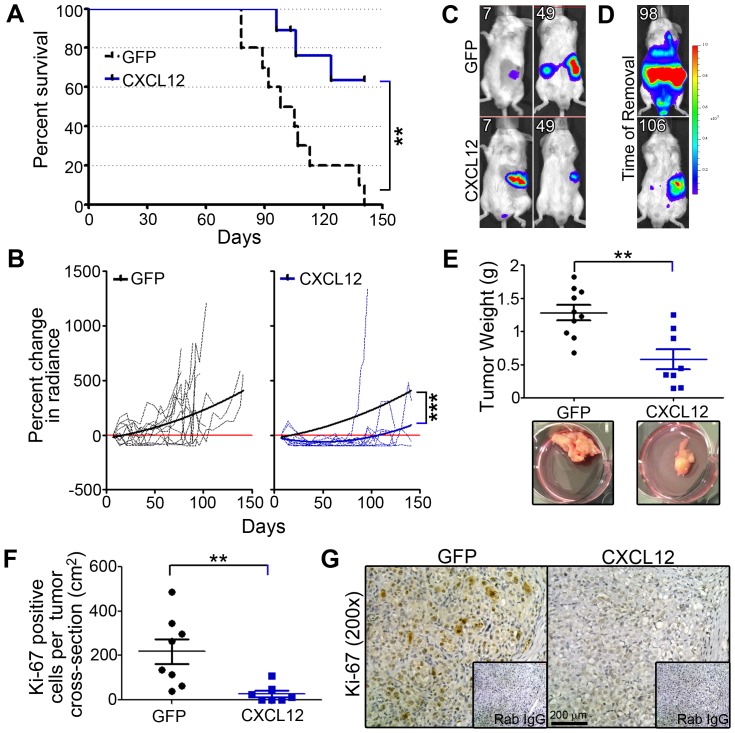
Autocrine CXCL12 delayed primary Autocrine CXCL12 delayed primary tumor growth and increased survival in an *in vivo* orthotopic pancreatic cancer model. (A) Kaplan-Meier survival curves for GFP- and CXCL12-expressing cell groups. Three experimental CXCL12 PDAC injected mice were removed for non-study reasons (tick marks). (B) Percent change in bioluminescence from baseline-level measured at day 7 for both GFP and CXCL12 engrafted mice. Dotted lines represent individual mice. Solid lines are quadratic regression fitted curves of each group. Statistical comparison was done between both groups independent of time. (C–D) Representative bioluminescence images of mice from each groupat days 7, 49, and endpoint for GFP (98) or CXCL12 (106). (E) Tumor wet weight was significantly reduced in CXCL12-expressing tumors relative GFP-tumors. Representative photomicrographs are shown in lower panels. (F) CXCL12-producing tumors had significantly fewer Ki-67 positive cells compared to GFP-expressing tumors, as counted in a cross-section of each tumor normalized to the total cross-sectional area of each tumor. (G) Representative images of Ki-67immunostaining and rabbit isotype control (inset, Rab IgG) are shown.n = 8–10. (**) and (***) denote *P*≤0.01 and *P*≤0.001 respectively, between CXCL12-expressing and control xenografted mice.

Finally, the *in vivo* functional role of CXCL12 in PDAC progression was modeled using an orthotopic xenograft technique. As expected, MiaPaCa2 luciferase cells consistently metastasized through hematogenous routes, with mice succumbing to metastatic tumor burden in a 90 to 140 day window (Figure 10). A survival study was conducted whereincontrol GFP or experimental CXCL12-secreting cellswere injected directly under the pancreatic capsule and tumor progression tracked using bioluminescence imaging.Overall, mice injected with CXCL12-expressing cells were found to have a significantly increased survival advantage compared to control mice (Fig.10A).While control mice had significanttumor burden by day 141, the majority of mice implanted with CXCL12-expressing PDACremainedalive, with little to no apparent tumor. The presence of CXCL12 increased survival, with a hazard ratio of 4.9. Weekly tracking measurements of total radiance in the animals revealed a significant decrease in percent change of tumor burden throughout the course of the study (Fig.10B).Over time, mice with tumor cells producing CXCL12 had lower tumor burden when assessed at early, middle, and later time points as visualized by bioluminescent imaging at days 7, 49, and 98, respectively (Fig.10C–D).

**Figure 11:**
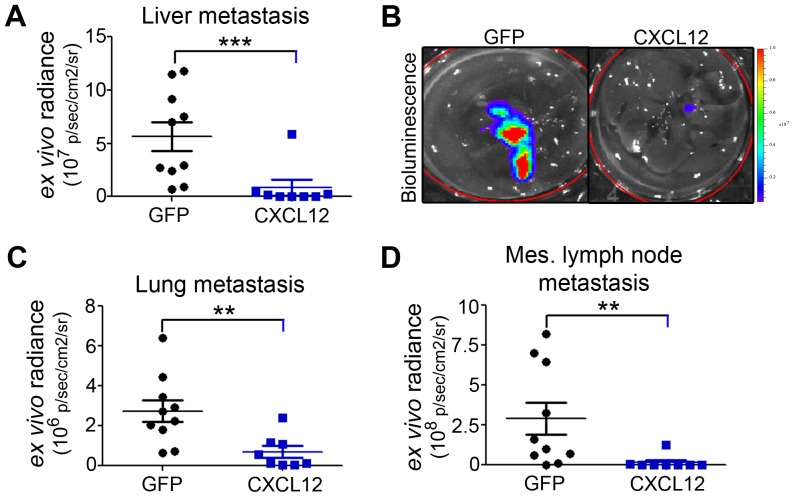
Distant metastasis in an orthotopic xenograft model of pancreatic cancer. *Ex vivo* bioluminescence analysis revealed significantly decreased metastasis to the liver (A), lung (C), and mesenteric lymph nodes (D) of CXCL12-expressing cells compared to GFP-controls. Representative biophotonic images of hepatic metastases are shown in panel B. (**) and (***) denote statistically significant *P*≤0.01 and *P*≤0.001, respectively, differences between CXCL12-expressing and control tumor engrafted mice. n = 8–10 mice in each group.

*Ex vivo* analysis confirmed that CXCL12 expression was a significant impediment to PDAC progression. Tumor wet-weight was significantly lower in mice with CXCL12 compared to GFP control cells (Fig.10E).Consistent with the delayed cell-cycle arrest observed *in vitro*, immunohistochemistry revealed that CXCL12, CXCR4, and CXCR7-expressing tumors had decreased focal expression of Ki-67 compared with control tumors, indicating lower proliferative potential *in vivo* (Fig.10F–G). Increased survival in mice with CXCL12-expressing PDAC was correlated not only with decreased primary tumor proliferation but also with the pronouncedabsence of tumor metastasis (Fig.11). Specifically, hematogenous metastasis to the liver and lung was significantly abrogated (Fig.11A–C), in addition todecreased lymphatogenous metastasis to the mesenteric lymph nodes (Fig.11D). Cumulatively, these *in vivo* data reflect the tumor suppressive potential of CXCL12 expression in PDAC cells.

## Discussion

Our findings demonstrate a seminal role for the homeostatic epithelial expression of CXCL12 in suppressing PDAC tumorigenesis. The conventional paradigm for chemokine involvement in malignancy stems from the correlation between elevated chemokine receptor levels, in particular CXCR4, and increased migration in cell culture [17]. The initial model suggested that cancer cells hijack CXCR4, allowing the malignant cell to follow endocrine gradients of ligand produced by distant tissues [38]. Subsequently, our work in both colorectal and breast cancer has shifted the perspective on the role of the CXCL12-CXCR4 axisin metastasis. We first established that autocrine expression of CXCL12 in normal intestinal and breast epithelial cells is epigenetically down-regulated by hypermethylation of the promoter region of its gene [24], [25].Retention of CXCR4 expression in CXCL12 silenced colorectal or breast cancer cells resulted in increased metastatic potential.With over 75% of patients diagnosed with either local or distant metastasis [57],PDACis a highly aggressive form of cancer in need of more effective therapies. Although CXCR4 expression has been previously correlated with human PDAC, we show here that human PDAC malignancy is halted following re-expression of its cognate ligand CXCL12.Specifically, CXCL12 interrupted both primary tumor growth, through cell-cycle arrest, and cancer cell metastasis, leading to increased overall survival in diseased animals. These inhibitory mechanisms are distinct from those we defined in colorectal or mammary carcinoma and suggest CXCL12 is a tumor suppressive cellular brake limiting PDAC malignancy.

Work from our laboratory was among the first to show a beneficial preclinical effect of autocrine CXCL12 on carcinoma malignancy [24]–[26], [43], [56]. Autocrine CXCL12 has similar pre-clinical effects in lung cancer progression [28] andhas been correlated with positive clinical outcomes in patients with osteosarcoma or breast cancers [31], [34]. In human colon carcinoma cells we determined that re-expression of CXCL12 restored sensitivity to detachment-induced apoptosis, a key molecular brake preventing dissemination of cancer cells [26], [43]. In contrast, in breast cancer we found that while autocrine CXCL12 decreased hematogenous spread of tumor cells, it also increased proliferation of the primary mammary fat-pad-engrafted tumor [25]. Shown here, human pancreatic cancer cells were strikingly different from colon and breast cancer in that re-introduction of autocrine CXCL12 expression decreased the growth and migration potential *in vitro*, while concomitantly decreasing growth and metastasis of PDAC cells *in vivo*.In contrast, there was minimal change in anoikis-sensitivity of CXCL12-expressing PDAC cells. Thus, CXCL12 appears to be broadly effective in limiting cancer progression, invasion, and metastasis and this benefit resultsfrom cell-type specific mechanisms. The tumor suppressive properties of CXCL12 may be translatable to the clinic as we have shown that administration of the recombinant protein provides an equally strong survival benefit in a preclinical colon cancer model [56].

Our study is the first to comprehensively study the expression of CXCL12, CXCR4, and CXCR7, a newly characterized member of the signaling axis, in normal and malignant pancreatic tissue. Congruent with its near ubiquitous organ expression [58], we found strong expression of CXCL12in normal pancreatic tissue, localized largelyto ductal epithelial cells. By contrast, CXCR4 and CXCR7 were evident in exocrine ducts, acini, and endocrine islets. Further, there was mixed expression of CXCL12, CXCR4 and CXCR7 in PanIN precursor lesions.These results suggest potential roles for CXCL12 and it receptors CXCR4 and CXCR7 in normal physiology. The exclusive expression of CXCL12 in normal pancreatic ductal epithelial cells, relative to acinar cells or endocrine islets, suggests the ligand may have homeostatic contributions to the exocrine pancreas.Further explorations into their physiologic roles in the pancreas, however, are hindered by embryonic lethality of CXCL12, CXCR4 and CXCR7 knockout mice [59]–[62]. Likewise, there is no established normal human pancreatic epithelial ductal cell line available for mechanistic studies *in vitro*.

CXCL12 expression was markedly absent, and CXCR4 and CXCR7 expression were more pronounced in human PDAC tumor cells. These data, usingour well-established technique for analyzing chemokine expression [24], [25],settles conflicting results between two previous studies, one that reported CXCL12 was over-expressed in human PDAC [35] and the other that showed that CXCL12 was absent in human PDAC tumor cells [36].Bothreports, however, were limited to malignant tissue and did not include the normal or benign tissue controls analyzed herein. Ourimmunostaining approach allowed for the discrimination betweenCXCL12, CXCR4, and CXCR7 expression levelswithin adenocarcinoma, tumor-associated fibroblasts, endothelium, and normal ducts and revealed a pronounced decrease in chemokine within the malignant ductal cell. Moreover, the histopathological decrease in CXCL12 observed in the tissues was recapitulated at the mRNA and protein level in several primary patient-derived as well as established PDAC cell lines. In their prior report,Liang and colleagues [35] used an immunostaining amplification kit to report elevated levels of CXCL12 in human pancreas cancer. Given the use of identical antibodies in both Liang and our studies, the resulting differences likely reflect the differing immunohistochemical staining procedures, in which the amplification kit likely overwhelmed detection limits for the constitutively produced and secreted CXCL12 protein. We additionally noted that CXCL12 expression appeared higher in tumor tissue from patients that had received neo-adjuvant therapy, a therapeutic regimen that improves survival in PDAC patients undergoing surgical resection [52]–[54]. It is tempting to speculate that the increase in CXCL12 expression reflects a direct effect of the gemcitabine and/or FOLFIRINOX treatment regimens. However, the effect of those drugs on CXCL12 transcript or protein expression was not directly assessed. Alternatively, the neoadjuvant treatment preferentially kills tumor cells lacking CXCL12, resulting in the observed increase in chemokine ligand expression.Our data support that of Zhong et al., and indicate that CXCL12 expression is pathologically diminished in human pancreatic cancer compared to healthy human pancreatic exocrine ductal epithelium.

Our work is the first to demonstrate that pancreatic cancer cells, like numerous other cell types, migrate towards exogenous sources of chemokine in a biphasic-concentration dependent manner [55], [56], [63]. As has been shown in previous *in vitro* experiments, exogenously administered concentrations of CXCL12 close to 10 nM stimulate chemotactic migration of cells through the monomeric form of the chemokine, while higher doses close 100 or 1000 nM lead to cytostasis through the predominant dimeric form of the chemokine. Endogenous or autocrine CXCL12 expression disrupted the ability of pancreatic cancer cells to migrate and home to distant sites of metastasis. Intriguingly, CXCL12 also altered non-migratory functions of pancreatic cancer cells, with stable re-expression of the liganddecreasing population growth via cell-cycle arrest *in vitro* and correspondingly smaller tumor xenografts with decreased proliferation markers *in vivo*. Importantly, re-introduction of CXCL12 resulted in a marked improvement in survival of tumor-bearing mice. The hazard ratio from the survival study was 4.9, reflecting the powerful effect CXCL12 expression imposed on altering the rate of tumor-induced mortality. These results in pancreatic cells suggest a tissue specific, tumor-suppressive role for CXCL12 that is adaptive to the unique programming of particular organs.Our data suggest that CXCL12 plays an integral role in the tumor progression which characterizes PDAC. In total, our studies reveal novel aspects of CXCL12 function in cancer biology and identify this gene as a multifunctional tumor suppressor that affects both PDAC growth and metastasis.
